# Co-evolution of SARS-CoV-2 variants and host immune response trajectories underlie COVID-19 pandemic to epidemic transition

**DOI:** 10.1016/j.isci.2023.108336

**Published:** 2023-10-27

**Authors:** Ranjeet Maurya, Aparna Swaminathan, Uzma Shamim, Smriti Arora, Pallavi Mishra, Aakarshan Raina, Varsha Ravi, Bansidhar Tarai, Sandeep Budhiraja, Rajesh Pandey

**Affiliations:** 1 Division of Immunology and Infectious Disease Biology, INtegrative GENomics of HOst-PathogEn (INGEN-HOPE) Laboratory, CSIR-Institute of Genomics and Integrative Biology (CSIR-IGIB), Mall Road, Delhi 110007, India; 2 Academy of Scientific and Innovative Research (AcSIR), Ghaziabad 201002, India; 3 Max Super Speciality Hospital (A Unit of Devki Devi Foundation), Max Healthcare, Delhi 110017, India

**Keywords:** Health sciences, Medicine, Virology

## Abstract

COVID-19 pandemic saw emergence of multiple SAR-CoV-2 variants. Exacerbated risk of severe outcome and hospital admissions led us to comprehend differential host-immune kinetics associated with SARS-CoV-2 variants. Longitudinal investigation was conducted through different time periods of Pre-VOC and VOCs (Delta & Omicron) mapping host transcriptome features. Robust antiviral type-1 interferon response marked Omicron infection, which was largely missing during Pre-VOC and Delta waves. SARS-CoV-2-host protein-protein interactions and docking complexes highlighted N protein to interact with HNRNPA1 in Pre-VOC, demonstrating its functional role for enhanced viral replication. Omicron revealed enhanced binding efficiency of LARP1 to N protein, probably potentiating antiviral effects of LARP1. Differential expression of zinc finger protein genes, especially in Omicron, mechanistically support induction of strong IFN (Interferon) response, thereby strengthening early viral clearance. Study highlights eventual adaptation of host to immune activation patterns that interrupt virus evolution with enhanced immune-evasion mutations and counteraction mechanisms, delimiting the next phase of COVID-19 pandemic.

## Highlights

• Omicron infection was marked by a robust type 1 interferon response
• SARS-CoV-2 N protein exhibited differential host protein interaction in VOCs infection
• Docking showed better viral replication in Pre-VOC whilst viral clearance in Omicron
• ZFPs in Omicron infection further supported its strong antiviral response

## Graphical abstract

**Figure:**
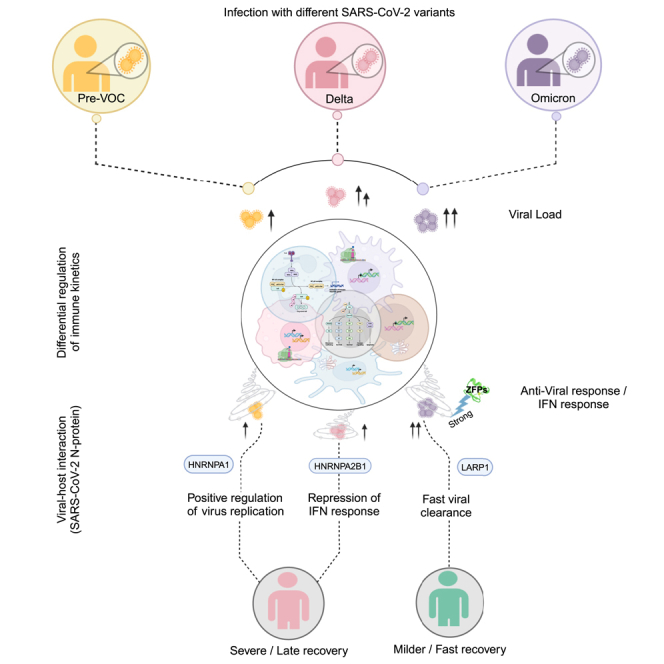

## Introduction

Since the identification of SARS-CoV-2 virus in humans in late 2019, the pandemic has caused over 6.9 million deaths globally, as of May 24, 2023 (https://covid19.who.int/). As the pandemic progressed, an increased risk of SARS-CoV-2 acquiring advantageous mutations resulted in the emergence of variant of concerns (VOCs), thereby potentially altering the transmissibility, disease severity, and escape from vaccine-induced/natural immunity.^1^ Notably, the variant that caused the first wave in India differed from the wild-type Wuhan strain by a D614G mutation in the spike and P232L in RNA-dependent RNA polymerase (RdRp) that made it more infectious and transmissible.^2^ Subsequently, the second wave caused by the Delta variant led to a widespread challenge including shortage of hospital beds, medications, and oxygenation support.^3^ Additionally, significantly higher number of females, younger age groups, and those without underlying comorbidities required ICU admission during the second wave as compared to the first wave.^4^ On the contrary, though the emergence of Omicron and its sub lineages showed greater transmissibility with reproductive number being 3.8 times higher than with the Delta, Omicron infected individuals displayed milder symptoms, decreased hospital stay and mortality.^5^ Overall, infections caused by the Omicron variant were observed to have a milder disease course. Besides viral evolution, it is imperative to comprehend the simultaneous evolution of host immune kinetics and its role in disease severity during different SARS-CoV-2 variant infections. A study by Laine et al. showed that the SARS-CoV-2 variants – Alpha, Beta, Delta, and Omicron – elicited a delayed and similar levels of interferon responses in infected human lung epithelial calu-3 cells.^2^ Conversely, Bojkova et al., demonstrated a reduced antagonism to interferon response by Omicron as compared to Delta, making the Omicron variant more sensitive to interferon response. This could possibly be related with the low inherent pathogenicity shown by the Omicron.^6^ Evidence by Alfi et al. showed that replication of Omicron is highly restricted in the lung tissue as compared to the other VOCs, whereas, it remained relatively unchanged in nasal tissue through *ex vivo* infection of human native nasal and lung tissues. They also reported a strong interferon response by Omicron, particularly in lung tissues, whereas the innate immune response was completely suppressed for Delta and other earlier VOCs.^7^ Several studies attributing to different levels of comparison between the SARS-CoV-2 variants in animal and cellular models have been reported. O’Donnell et al. compared transcriptomic differences of Alpha and Beta against the wild-type SARS-CoV-2 by inducing the infection in Syrian golden hamsters and observed that Alpha induced a more robust inflammatory response.^8^ A study highlighted enhanced pathology and inflammatory response against the Delta infection in mice as compared to the Alpha^9^ Additionally, Kuruppuarachchi et al. reported that wildtype variants exhibited the highest pathogenicity followed by Delta and then Omicron in transgenic mice.^10^ Thus, we see different variants elicit different degrees of immune response, antibody evasion as well as different pathogen characteristics across the SARS-CoV-2 variants.

Additionally, infection dynamics of SARS-CoV-2 and the antigenic shifts to evade host immunity are highly dependent on the host characteristics that influence the selection pressure within an endemic setting.^11^ Xu et al. had highlighted the sensitivity of Omicron variant to interferons emphasizing reduced viral shedding in response to Interferon-α2b (IFN-α2b) spray in a Chinese cohort.^12^ Thus, it can be deliberated that the global transmission of similar SARS-CoV-2 variants at any given time period with generalized clinical manifestations and outcome might point toward application of population-based findings worldwide. Nonetheless, its adaption to specific populations could also influence their characteristics, which in turn can maneuver the host response mechanisms and outcome. Taken together, it is important that the immune trajectories associated with different SARS-CoV-2 variants, particularly VOCs needed localized studies to strengthen the global understanding.

Here, in our study, we aim to comprehensively understand the transcriptome signature, particularly the immune landscape exhibited by the patients infected with different SARS-CoV-2 variants in India. Nasopharyngeal swabs from 211 COVID-19 positive hospital-admitted patients were collected to analyze the initial infection stage host mRNA expression presentation, further corroborated with the clinical presentation to gain insights into the immune kinetics of Pre-VOC, Delta, and Omicron. Further, to delve deeper into the effect of mutations possessed by different variants of SARS-CoV-2, host-pathogen protein-protein interaction analysis was performed for each variant. Study insights revealed the induction of a robust antiviral interferon response to Omicron infection which was largely missing during the Pre-VOC and subsequent VOC surges. Moreover, with passage of time, the mutations which were known to confer an edge to virus transmission and infectivity might be possibly aiding the host to reverse/halt the viral infection, an important direction toward diminishing viral evolution and thus the pandemic.

## Results

### Study design and patient clinical characteristics of Pre-VOC, Delta, and Omicron

**Figure 1:**
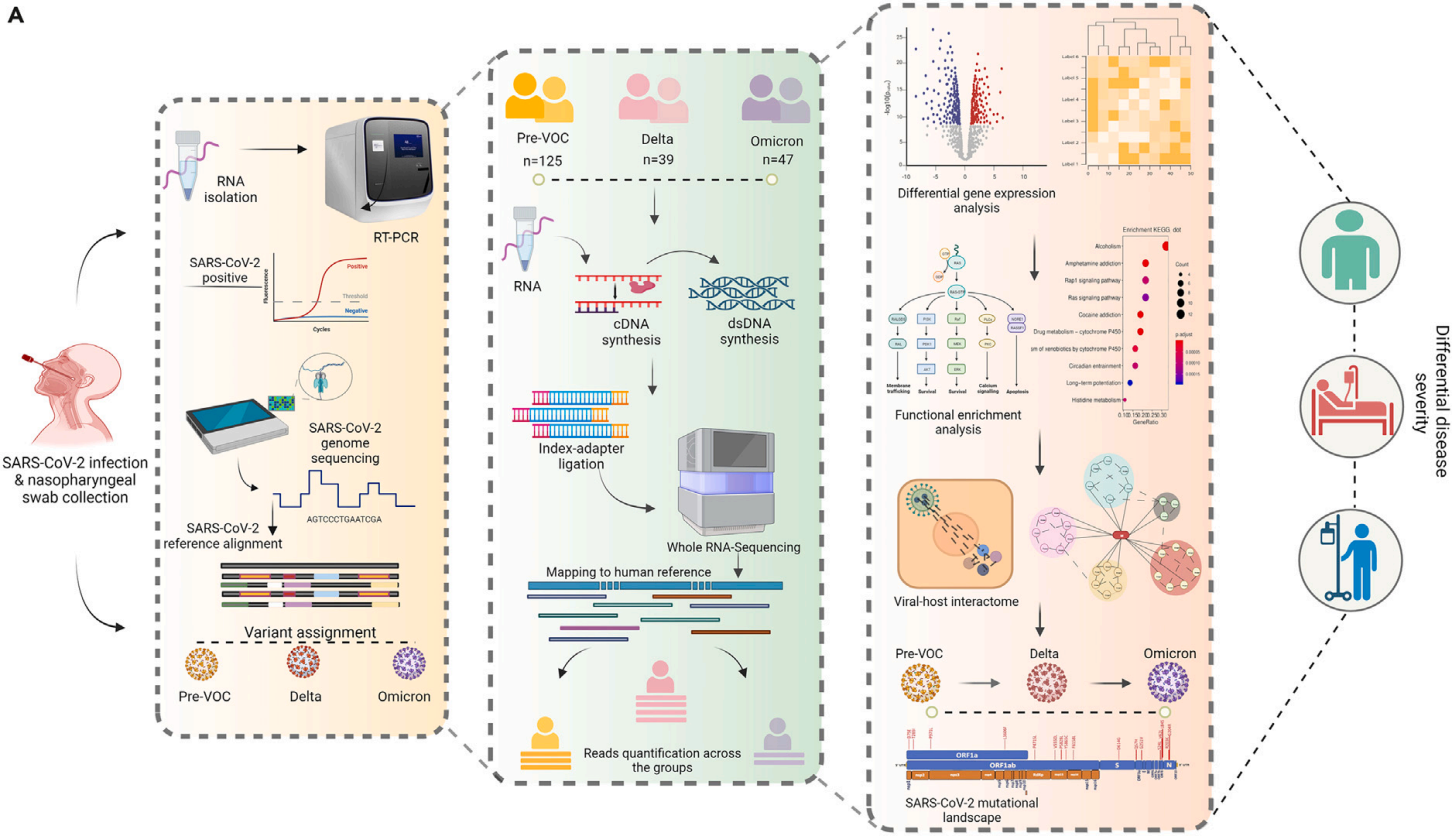

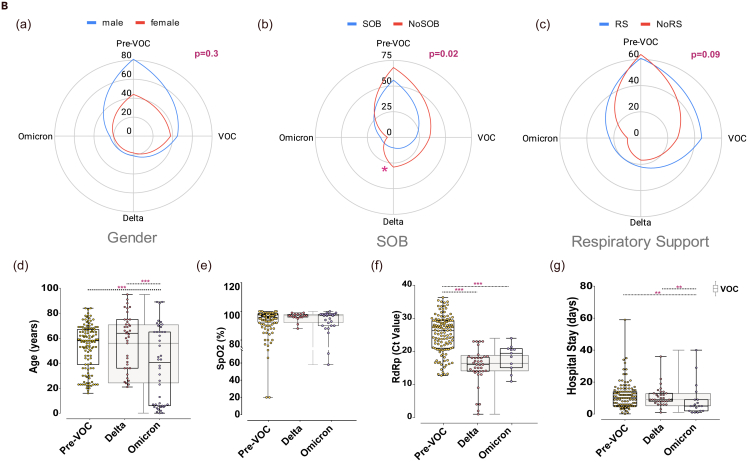
Study design and patient clinical characteristics (A) Overview of experimental workflow depicting study design. Highlights the cohort of hospital-admitted patients infected with Pre-VOC, Delta and Omicron, identified through SARS-CoV-2 genome sequencing. Human host transcriptomic data analysis followed by analysis toward differentially expressed (DE) genes, downstream functional analyses, visualizations and study outcome. (B) Spider plot capturing clinical parameters for Pre-VOC, Delta and Omicron infected COVID-19 patients. (a) Gender, (b) SOB, (c) respiratory support requirement, (d) Age, (e) SpO2 levels, (f) Ct value of SARS-CoV-2 *RdRp* gene, and (g) Duration of hospital stay, with statistical significance measured using Mann-Whitney *U* test (‘∗’, ‘∗∗’, ‘∗∗∗’ signifies p *value* of <0.05, <0.01, <0.001 respectively).

This study aims to understand the diversified host response to infection with different SARS-CoV-2 variants, Pre-VOC and VOCs (Delta and Omicron). The study cohort of 211 hospital-admitted COVID-19 patients was a subset of a large number of samples collected during the genome surveillance program of SARS-CoV-2 carried out starting March 2020 till date. During this period, India witnessed notable waves due to surge in COVID-19 cases by emergence and infection with different SARS-CoV-2 variants at distinct time points. The COVID-19 positive patients confirmed by RT-PCR underwent SARS-CoV-2 genome sequencing for SARS-CoV-2 variants identification. A total of 211 patients with detailed clinical data were segregated based on infection with different SARS-CoV-2 variants. Patients were categorized into two groups – (1) Pre-VOC (n = 125) between April–July 2020, and (2) VOCs (n = 86) which included Delta and Omicron patients, March-April 2021 (Delta) and Jan–March 2022. Within the VOC group, patients infected with Delta (n = 39) and Omicron (n = 47) were segregated to study the host response with respect to specific VOCs. Bulk RNA-seq performed to elucidate landscape of host transcriptome yielded 2,426,360,877 reads with an average of 114,993,401 reads across the samples after a stringent quality check. Differential gene analysis was carried out between the groups (1) Pre-VOC and VOCs, (2) Delta and Omicron, (3) Pre-VOC and Delta, and (4) Pre-VOC and Omicron, to obtain a comprehensive understanding of dynamic host transcriptome altered with exposure to different SARS-CoV-2 variants. Pathway enrichment analysis and viral-host interactions were investigated to provide insights for different clinical manifestations associated with Pre-VOC, Delta, and Omicron. Figure 1A illustrates the study design, methodology, and downstream analysis.

Next, we evaluated the demographics and clinical features of the patients infected with Pre-VOC, Delta, and Omicron variants. Table 1 demonstrates the number of patients in each variant group with varying clinical features, as well as providing the missing data information. Taking into account those patients with available clinical data, we observed that the percentage of males and females were comparable in Delta (M/F = 53.8/46.1) and Omicron (54.5/45.4), whereas the proportion of males was higher in Pre-VOC (64.5/35.4). The median age of patients in Omicron (40.5 years) was significantly lower than Pre-VOC (58 years) and Delta (64 years) (p *value=0*.*003*). Notably, children (below 12 years) were primarily seen in the Omicron whereas the same was not observed for Pre-VOC and Delta. The viral load was high in Delta with lower Ct value of 16 for *RdRp* gene as compared to Pre-VOC (Ct value 26). The *RdRp* gene Ct value for the Omicron group was available for n = 11 patients only, which also reflected a similar trend of low Ct value as Delta. Shortness of breath (SOB) was a distinctive feature of Pre-VOC as higher number of individuals showed SOB, although median SpO2 levels did not differentiate between the variant groups. The patients who required respiratory support were higher in Delta (56%) followed by Pre-VOC and Omicron. Furthermore, the recovery rate in Delta was the lowest (41%) as compared to Omicron (59.5%) and Pre-VOC (85.6%) suggesting that Delta had a comparatively severe clinical presentation. The presentation of the clinical features across Pre-VOC, Delta, and Omicron is depicted in Figure 1B.

**Table 1:** Demographic and clinical parameters

Demographic parameters	Pre-VOC (n = 125)	Delta (n = 39)	Omicron (n = 47)	p values
Gender Male/Female	80/44	21/18	24/20	0.331^a^
Missing	1	0	3	
Age (years), median IQR	58 (16–84)	64 (21–95)	40.5 (0.07–89)	0.003^b^
Missing	1	0	3	
Ct value (*RDRP* gene)	26.2 (12.7–36.3)	16 (1–23)	19.4 (10.9–24.0)	<0.001^b^
Missing	1	2	36	
Shortness of breath (n;%)	56 (44.8)	10 (25.6)	12 (25.5)	0.006^a^
Missing	11	0	29	
SpO_2_ levels, median IQR	96 (0–100)	97 (88–99)	97 (58–100)	0.653^b^
Missing	11	23	20	
Respiratory support (n;%)	61 (48.8)	22 (56.41)	23 (48.9)	0.455^a^
Missing	0	0	8	
Hospital stay (days), median IQR	10 (0.1–59)	9 (1–36)	5 (1–40)	<0.001^b^
Missing	2	7	28	
Recovery rate	85.6	41.0	59.6	<0.00001^a^
Missing	0	0	0	
Vaccination	0	16	12	<0.001^a^
Missing	0	23	30	

Summarized data across the Pre-VOC, Delta and Omicron.

a Chi-Square.

b Kruskal-Wallis.

### Early heightened antiviral interferon response delineates Omicron infection from Delta and Pre-VOC with fast recovery and mild presentation

**Figure 2:**
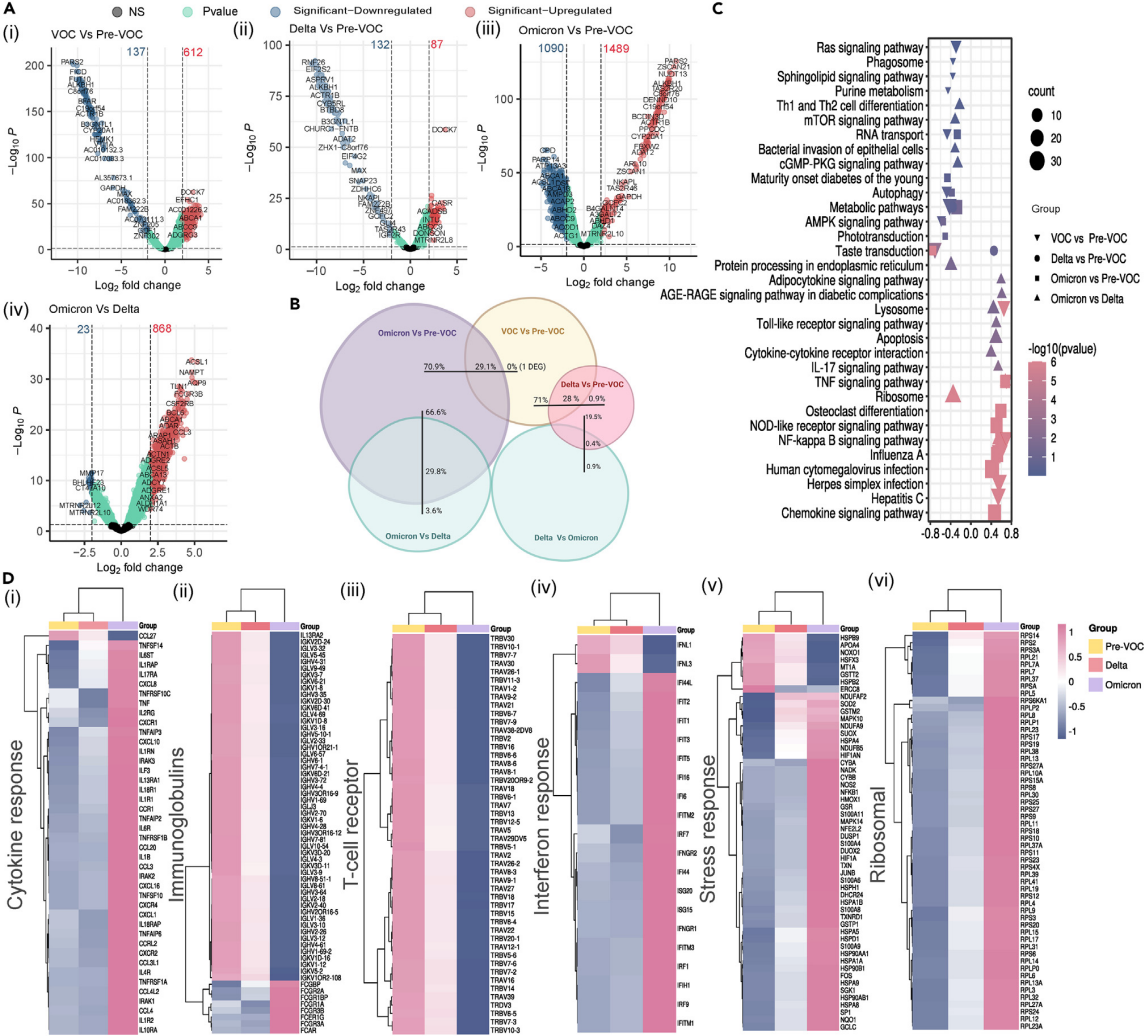
Differential expression, functional enrichment and comparative normalized counts of DEGs across Pre-VOC, Delta, and Omicron (A) Volcano plot representing DEGs with log2 fold change of ±2 and adjusted p *value* < 0.05 in (i) VOC vs*.* Pre-VOC, (ii) Delta vs*.* Pre-VOC, (iii) Omicron vs*.* Pre-VOC, and (iv) Omicron vs*.* Delta. (B) Conceptual visualization of DEGs distribution. (C) GSEA analysis of DEGs. (D) Heatmap representations of distinct family of DEGs, (i) Cytokine response, (ii) Immunoglobulin receptors, (iii) T cell receptors, (iv) Interferon response, (v) Stress response, and (vi) Ribosomal proteins.

The first tier of differential gene expression analysis was performed between the Pre-VOC and VOCs to obtain a comprehensive host transcriptome map pertaining to infection with VOCs that triggered an excessive surge of infections in India. Analysis yielded 749 significant differentially expressed genes (DEGs), with 612 upregulated and 137 downregulated genes in the VOCs (Figures 2A-i). The upregulated genes in VOCs spanned over vastly different family of genes responsible for heat shock response, interferon-induced response, chemokines, interleukins, tumor necrosis factors (TNF), major histocompatibility complexes (MHC), ribosomal protein genes, and NF-kB signaling. Next, we assessed the DEGs profile within the VOCs, i.e., Omicron vs. Delta, which captured 891 significant genes wherein 868 were upregulated and 23 were downregulated in Omicron (Figures 2A-iv). Interestingly, the majority of the upregulated genes in the Omicron fell in the same family of genes as observed in the VOCs.

Nonetheless, this differential host gene regulation by VOCs overall and striking differences within VOCs as well allowed us to delve into VOC-specific responses. Whilst Delta vs*.* Pre-VOC demonstrated 219 significant DE genes in total, Omicron vs*.* Pre-VOC displayed 2579 significant DE genes. Notably, 1489 genes were upregulated and 1090 genes were downregulated in Omicron vs*.* Pre-VOC deliberating a remarkable change in host transcriptome induced by the Omicron (Figures 2A-iii). On the other hand, only 87 genes were upregulated and 132 genes were downregulated in Delta vs*.* Pre-VOC (Figures 2A-ii) suggesting sub-optimal host response in Delta. Moreover, the DEGs observed in Omicron vs*.* Pre-VOC and Delta vs*.* Pre-VOC covered nearly all the genes reportedly present in VOCs vs*.* Pre-VOC analysis thereby reflecting the differential expression of similar families of genes (Figure 2B). However, intriguingly, the genes of immunoglobulin receptors, T cell receptors and keratin proteins were present additionally and exclusively in Omicron vs*.* Pre-VOC comparison. Thus, the differential gene expression analysis across these groups indicate a differential host transcriptome in response to VOCs overall as well as by specific VOCs, Delta and Omicron, with Omicron infection inducing vast and rigorous host response.

Subsequently, gene set enrichment analysis (GSEA) was performed on the DEGs to highlight the enriched pathways across the variant groups (Figure 2C). We captured gene signature related to diseases like influenza A, Herpes simplex, cytomegalovirus, hepatitis which signified that our gene set from different variant groups were highly related to different viral infections. Moreover, several immune and inflammation-related signaling pathways such as, TNF, NFkB, AGE-RAGE, NOD-like receptor, TOLL-like receptor, adipocytokine, IL-17, Chemokine, cytokine receptor interaction, RAS, mTOR, and AMPK signaling were seen in VOCs vs*.* Pre-VOC, Omicron vs*.* Pre-VOC, and Omicron vs*.* Delta, signifying heightened antiviral and innate immune response especially in Omicron. Ribosome and protein processing pathways were essentially seen in Omicron vs*.* Delta.

Figure 2D demonstrate the heatmaps of normalized gene count for DEGs modulating the host transcriptome landscape in response to infection across the study groups. The DEGs were divided into different categories based on their possible functional role in disease regulation. Remarkably, the interferon induced genes/interferon response genes family was observed to have a higher expression in Omicron when compared to Pre-VOC and Delta (Figure 2D-iv). IFITs (Interferon-induced proteins with tetratricopeptide repeats) and IFITMs (Interferon-induced transmembrane proteins) are first line defenders which restrict viral replication/translation as well as host cell entry. Along with interferon regulatory factors (IRFs) and interferon-stimulated genes (ISGs), Type 1 interferon response seems to be dominant in Omicron whereas higher expression of *IFNL1* and *IFNL3* in Pre-VOC suggests Type 3 interferon response. It has been reported that Type 1 IFN responses were most significantly enriched in patients with mild (compared to severe or critical) COVID-19.^13^ Similarly, the genes involved in stress response, cytokine response and large number of ribosomal proteins were also seen to have a higher expression in the Omicron as compared to Delta and Pre-VOC (Figure 2D-i,v,vi). In contrast, T cell receptor (TCR) and B cell receptor (BCR) genes showed lowest expression in Omicron followed by Delta and highest in Pre-VOC (Figure 2D-ii,iii). Interestingly, it seems probable that BCR and TCR clonal expansion is observed to be different and higher after primary SARS-CoV-2 infection, which is the case for Pre-VOC and reduced after subsequent reinfection or vaccination which reflects in Delta and Omicron. Moreover, Fcγ receptors and IgG Fc-binding protein (Fcγbp), both are upregulated in Omicron which again confers to antiviral activity and clearance, responding to milder phenotype. The findings taken together collectively indicate a robust induction of early central antiviral response i.e., Interferons specific to Omicron thereby promoting innate immune response via cytokines and antigen presentation and at the same time activated mechanisms for viral clearance, corresponding to mild presentation and quick recovery in Omicron.

### Unique viral-host interactions in Omicron, Delta, and Pre-VOC functionally segregates host response pathways for distinct SARS-CoV-2 variant’s N protein

**Figure 3:**
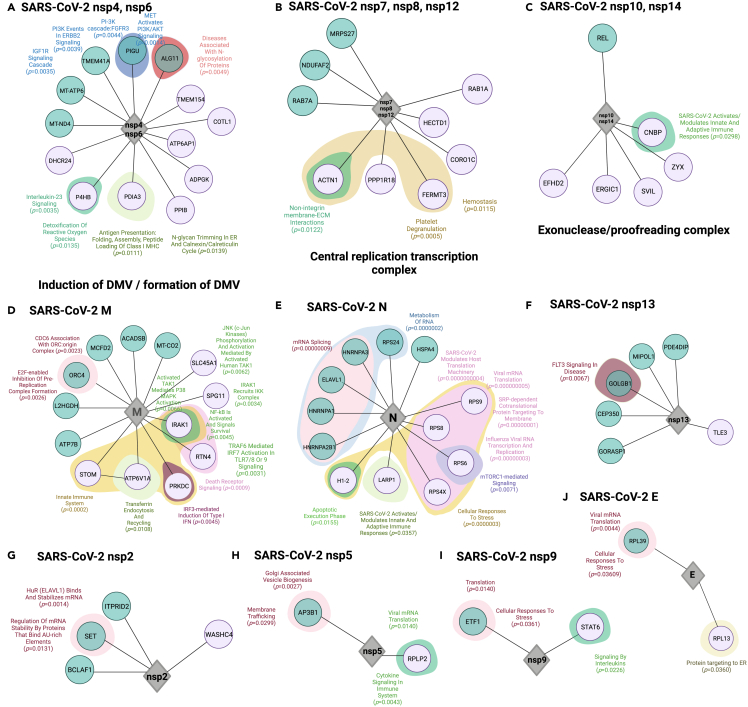
SARS-CoV-2 host unique protein-protein interactome Depicted across Pre-VOC vs. VOC (Green) and Omicron vs. Delta (pink) along with functionally enriched pathways for host proteins interacting with specific SARS-CoV-2 proteins. (A) nsp4-ns6 complex, (B) nsp7-nsp8-nsp12 complex, (C) nsp10-nsp14, (D) membrane, M, (E) nucleocapsid, N, (F) nsp13, (G) nsp2, (H) nsp5, (I) nsp9, and (J) envelop, E.

To comprehend the dynamic host immune response kinetics vis-à-vis SARS-CoV-2 variants, we analyzed viral and host protein–protein interactomes. It is indispensable for the virus to interact with the host proteins for their transcription and translation post entry for successful viral replication. Therefore, to elucidate the differential behavior of distinct VOCs and Pre-VOC, the DEGs obtained from our study groups’ comparison – (1) Pre-VOC vs*.* VOC and (2) Omicron vs*.* Delta – were taken as SARS-CoV-2 interactors. The SARS-CoV-2 host protein-protein analysis demonstrated a total of 118 interactions for Pre-VOC vs*.* VOCs and 137 interactions for Omicron vs*.* Delta. The observance of partially overlapping yet differential host transcriptome response amongst the variant groups led to the identification of 68 overlapping interactions across the groups (Table S1), whilst 50 and 69 unique interactions were captured in Pre-VOC (vs*.* VOC) and Omicron (vs*.* Delta) respectively (Table S2). Moreover, to analyze the biological functions of host proteins interacting with the SARS-CoV-2 proteins, we performed pathway analysis on the obtained SARS-CoV-2 protein interactome using Enrichr, a web-based tool. Figure 3 represents the SARS-CoV-2 and host protein-protein interactome and their associated host biological pathways for Pre-VOC and Omicron. Among the unique interactions across the two groups, the SARS-CoV-2 proteins – orf3a, Membrane (M), Nucleocapsid (N) and ORF7b, were observed to have the highest number of interactions, whereas the proteins – orf10, orf9c, orf7a, nsp9, nsp5, orf3b and Envelop (E) were observed to have least number of interactions.

The merging of individual nsps into their functional complexes yielded a total of 3 complexes in our study – (1) Double membrane induction by nsp4-nsp6 complex, (2) central replication-transcription complex by nsp7-nsp8-nsp12 complex, and (3) exonuclease/proofreading activity by nsp10-nsp14 complex. Notably, across these complexes, the nsp4-nsp6 complex showed interaction with *MT-ATP6*, *MT-ND4* (Figure 3A) and nsp7-nsp8-nsp12 complex with *NDUFAF2* (Figure 3B) in Pre-VOC respectively. Remarkably, these genes are involved in oxidative phosphorylation and assembly of ETC complex 1.^14^^,^^15^ On the other hand, nsp4-nsp6 interacted with the genes *P4HB*, *PDIA3*, *ATP6AP1* (Figure 3A) and nsp7-nsp8-nsp12 interacted with *RAB1A* gene in Omicron respectively (Figure 3B). It is important to highlight that *PDIA3* is important for antigen presentation during viral infections as reflected in the pathway.^16^^,^^17^ Also, *RAB1A* is involved in the vesicular transport and membrane trafficking pathway in the host. Subsequently, the analysis of nsp10-nsp14 proof reading complex demonstrated an interaction with CNBP host protein in Omicron (Figure 3C). Strikingly, CNBP is reported to have a strong interaction with the SARS-CoV-2 RNA eliciting a heightened antiviral response and its insufficiency could increase the host susceptibility to infections.^18^ It was observed that M is one of the SARS-CoV-2 proteins that show higher number of interactions with the host. M protein was primarily showing interactions that were essentially involved in innate immune system pathways such as NF-KB activation and signal survival, IRF3 mediated induction of type 1 interferons, death receptor signaling and was particularly abundant in Omicron (Figure 3D). In addition to M, the ORF proteins – orf3a and orf 7b were also interacting with genes enriched in innate immune pathways (Figure S1). Taken together, presence of a remarkable abundance of innate immune pathways exhibited by Omicron SARS-CoV-2 interactors indicate toward an extensive modification of host immune response in Omicron as compared to the previously emerged variants – Pre-VOC and Delta.

In addition to innate immune response genes seen to be enriched and interacting with SARS-CoV-2 proteins specifically in Omicron, we noticeably observed a differential interaction regulation of N protein in Pre-VOC vs*.* VOCs and Omicron vs*.* Delta. The N protein exhibited interactions with genes of mRNA splicing and metabolism – *HNRNPA3*, *ELAV1*, *HNRNPA1*, *HNRNPA2B1*, *RPS4*, and *HSP4* in Pre-VOC vs*.* VOCs whereas it was seen to interact majorly with ribosomal proteins – *RPS9*, *RPS8*, *RPS6*, *RPS4X* and the proteins *LARP1* and *H1-2* in Omicron vs*.* Delta (Figure 3E). Interestingly, HNRNPA1, which is a SARS-CoV-2 interactor in Pre-VOC group, has been reported to interact with N6-Methyladenosin (M6A) marked SARS-CoV-2 RNA for early translation to replication switch.^19^^,^^20^ In addition, the ELVAL1, a unique interactor of N protein in Pre-VOC, is RNA binding protein and reported to bind with SARS-CoV-2 RNA, thereby increasing the RNA stability.^21^ Additionally, SARS-CoV-2 nsp2, nsp5, nsp9, nsp13 and E showed least interactions as depicted in Figures 3F–3J. Furthermore, the overlapping SARS-CoV-2 – host protein-protein interactions were observed to be less diverse as compared to the unique interactions. Expectedly, the overlapping interactions showed pathways like interleukin signaling, NF-KB signaling, highlighting the activated immune response to SARS-CoV-2 by the host post infection (Figure S2).

### Docking analysis with N protein of Pre-VOC, Delta, and Omicron demonstrates strong antiviral mechanisms in Omicron patients

Host-pathogen interactions are modulated by mutations acquired by the pathogen throughout the course of its evolution. Therefore, to investigate the binding efficiency of different SARS-CoV-2 variant proteins carrying distinct mutations that interact with the host protein, molecular docking was performed. Notably, Nucleocapsid (N) protein and its interactors were taken forward due to its distinct interactions with host proteins that regulate SARS-CoV-2 replication complemented with the documented role of N protein in modulation of IFN response in the human host. Subsequently, the primary unique mutations analysis across the three groups – Pre-VOC, Delta, and Omicron – revealed a total of 2200 mutations, out of which 114 mutations demonstrated statistical significance through Fisher’s test (Table S3). The statistically significant N protein mutations across the three groups were further selected for docking analysis. We used HADDOCK to perform the protein-protein docking of wildtype and the mutant N proteins across Pre-VOC (S194L), Delta (R203M, G215C, D377Y), and Omicron (P13L, R203R, G204R, S413R) with its interacting host proteins (Table 2).

**Table 2:** Protein-protein docking of wildtype and mutant N protein with host proteins

Protein	Wildtype	Pre-VOC	Delta	Omicron
ELAV1	−11.4	−9.3	−10.9	−8.9
HNRNPA3	−11.1	−12	−11.1	−11.5
HNRNPA1	−14.1	−15.1	−14.4	−12.7
HNRNPA2B1	−8.9	−12	−12.4	−11.9
RPS4X	−11.1	−10.7	−9.6	−10.3
RPS6	−9.8	−10.3	−10.1	−13.3
RPS8	−12.9	−11.2	−13.6	−12
RPS9	−12.2	−12.1	−12.5	−11.8
HSPA4	−11.4	−14.3	−11.7	−15.4
RPS24	−12	−12.8	−12.2	−12.1
H1-2	−11.1	−10.6	−11.6	−9.7
LARP1	−10.9	−10.8	−10.1	−12.3

Binding energy (kcal mol-1) of host proteins and N protein docking complexes.

**Figure 4:**
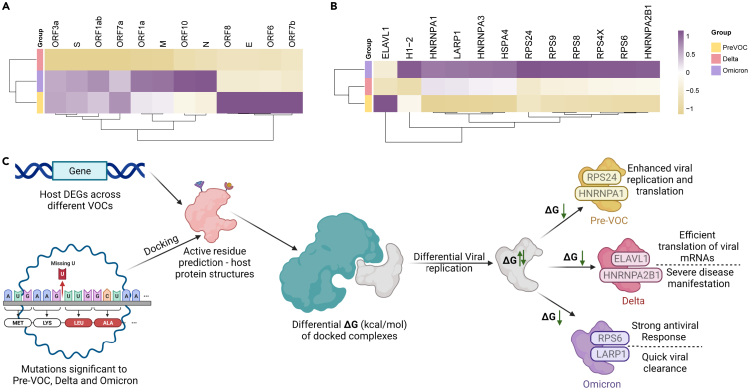
Conceptual visualization of SARS-CoV-2 Host protein–protein docking in Pre-VOC, Delta, and Omicron Heatmaps depicting (A) SARS-CoV-2 normalized gene counts. (B) Host DEGs, and (C) illustrations of docking interactions of N protein with host genes with differential binding efficiency leading to differential host response in Pre-VOC, Delta and Omicron.

The analysis of SARS-CoV-2 reads from our RNA-seq data demonstrated highest number of read counts for the N gene in Omicron, followed by Delta and Pre-VOC (Figure 4A). The docking performed for the Omicron N protein showed lowest binding energy with host proteins – LARP1 (−12.3 kcal mol-1), HSPA4 (−15.4 kcal mol-1), RPS6 (−13.3 kcal mol-1) as compared to the wildtype, Pre-VOC, and Delta. A strong and stable binding of Omicron N protein with the RPS6 protein indicates the viral hijack of translational machinery for its replication, thereby indicating a higher replication and transmission of the virus. However, on the other hand, its stable binding with LARP1 counteracts the viral replication by producing a strong antiviral response^18^ that could aid in quick viral clearance, given both the host proteins LARP1 and RPS6 are highly enriched in the Omicron group (Figure 4B) with comparatively higher N gene count, resulting in positively regulated interactions. Furthermore, Delta N protein showed good binding energy with majority of the host proteins – H1-2 (−11.6 kcal mol-1), RPS9 (−12.5 kcal mol-1), RPS8 (−13.6 kcal mol-1), HNRNPA2B1 (−12.4 kcal mol-1), ELAVL1 (−10.9 kcal mol-1) as compared to Pre-VOC and Omicron. This could be due to the specific presence of the mutation – G215C in the active site of Delta. Notably, the efficient binding of Delta N protein with HNRNPA2B1 could result in the repression of IFN response as HNRNPA2B1 is reported as a positive upstream regulator of IFN pathway.^22^ Collectively, a moderate expression of HNRNPA2B1 combined with the moderate SARS-CoV-2 N gene counts was observed (Figure 4C). Moreover, for the Pre-VOC N protein complexes, HNRNPA3 (−12 kcal mol-1), HNRNPA1 (−15.1 kcal mol-1), RPS24 (−12.8 kcal mol-1) showed highest binding energy as compared to the wildtype and the other two study groups. Notably, HNRNPA1 has been demonstrated to enhance viral replication upon binding to N protein, thereby inducing an early translation to replication switch. Additionally, its stable binding with the ribosomal protein RPS24 could suggest the viral usage of host machineries for translation. It is important to note that though the N gene demonstrated relatively much lower read counts in the Pre-VOC group (Figure 4A), it still could have an enhanced viral replication and translation pertaining to the group’s own clinical presentation and disease progression.

### Distinct expression of multiple ZFP (zinc finger protein) genes across Pre-VOC, Delta, and Omicron reveals their putative roles in modulating viral-host interactions

**Figure 5:**
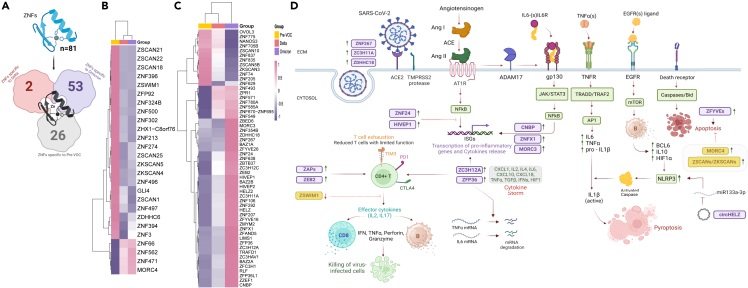
A schematic representation of the putative interplay of zinc finger genes with different immune signaling pathways (A) Visualization of ZFPs distribution across Pre-VOC, Delta and Omicron. Heatmaps depicting (B) Pre-VOC specific ZFPs (C) Omicron specific ZFPs. (D) Role of ZFPs in Omicron: *ZDHHC18*, *ZNF267*, and *ZC3H11A* augment viral entry and infectivity. *ZNF24*, *HIVEP1*, *ZC3H12A*, and *ZFP36* inhibit NFkB signaling and pro-inflammatory response. ZAPs and ZEB2 are positive regulators of T cell proliferation and adaptive immune response. *circHELZ* and *ZFYVEs* regulate pyroptosis and apoptosis. Role of ZFPs in Pre-VOC: *MORC4* and *ZSCANs/ZKSCANs* are known to inhibit apoptosis augmenting pathogenicity. The zinc finger genes specific for Omicron and Pre-VOC are represented in purple and yellow respectively (Created with licensed version of BioRender).

Consequent to analyzing viral-host interactions, we investigated distinct group of DE genes which were remarkably present across all variant groups and also known for their function to substantially engage in host-virus interplay during infection. The zinc finger protein (ZFP), having the capacity to bind both to viral genomes and host mRNAs, affects viral replications and host cell transcription, leading to antiviral/proviral immune responses. Our study captured 81 DE zinc finger genes across subgroups, of which 53 were unique to Omicron (present in Omicron vs*.* Pre-VOC, Omicron vs*.* Delta) and only 2 were found unique in Delta (Delta vs*.* Pre-VOC, Delta vs*.* Omicron). 26 ZFP genes were found specifically associated with Pre-VOC as was observed in both Omicron vs*.* Pre-VOC and Delta vs*.* Pre-VOC Figure 5A. A heatmap was constructed to analyze the expression of ZFPs in different variant groups.

Figure 5B demonstrates the Pre-VOC specific ZFPs, where out of 26 DE ZFPs, 22 ZFPs showed highest expression in Pre-VOC. Similarly, Figure 5C captures the expression of 52 ZFPs, which were differentially expressed in Omicron, with 41 showing upregulation and 10 downregulation and only one gene, *MORC3* was found upregulated in Omicron (vs. Delta). Further, of these 26 DE ZFPs specific to Pre-VOCs, 7 ZFPs (*ZKSCAN5*; *ZSCAN1*,*18*, *21*, *22*, *24*; *ZNF496*) are known to elicit inflammatory immune response^23^^,^^24^ whereas *ZSWIM1* is found to be directly involved in regulating T cell mediated viral clearance.^25^ Another ZFP expressed in Pre-VOC, *ZDHHC6*, facilitates palmitoylation of S required for syncytia formation, hence critical for SARS-CoV-2 entry.^26^ The upregulation of functionally distinct ZFPs in Pre-VOC indicate their dual role in conferring antiviral response as well as virulence, thereby conferring moderate clinical manifestations as observed in Pre-VOC. *ZKSCAN3* and *ZNF629* were differentially downregulated only for Delta. *ZNF629* is known to cause immunodeficiency and *ZKSCAN3* is a master repressor of autophagy.^27^ SARS-CoV-2 infection activates cell apoptosis leading to inflammatory cytokine processing and release through virus-induced necroptosis for enhanced severity.^28^^,^^29^ Hence, downregulation of these ZFPs might be effective in causing the infection to persist, as was observed in Delta infected patients, impacting disease severity. Likewise, of 52 DEGs unique to Omicron, 3 ZNFs (*ZNF267*, *ZDHHC18*, *ZC3H11A*) contributed to virulence in Omicron whilst a huge number of 18 ZFPs were involved in viral clearance including ZAP members known as interferon-inducible genes. These included *ZC3HAV1*, *HELZ*, *HELZ2*, *ZC3H12C*, *ZFC3H1*, *ZC3H12A*, *ZFP36*, and *ZFP36L1*, eliciting strong antiviral immune response. Moreover, the only ZNF found upregulated in Omicron (vs. Delta), *MORC3* is known to elicit antiviral response, although it has a complex and nuanced role in conferring inflammation as well. These findings highlight robust viral clearance in Omicron infection compared with Delta and Pre-VOC. Figure 5D summarizes the putative interplay of ZFP genes with immunological modulations, essentially in Omicron.

Briefly explaining, figuratively, the initiation of infection with SARS-CoV-2 using ACE2 as a receptor for viral entry and infectivity is augmented by ZFPs like *ZDHHC18*, *ZNF267*, and *ZC3H11A* (seen in Omicron infection). This might support the high viral load observed in the Omicron infected patients compared to the Pre-VOC, wherein Omicron showed a significantly lower Ct value of 19 whilst Pre-VOC showed Ct value of 26. Subsequently, it initiates ACE2 deficiency and ADAM17 activation, leading to induction of pro-inflammatory pathways. Explaining stepwise, with persistent Spike protein binding and shedding by ADAM17, ACE2 receptor gets depleted. Normally, ACE2 receptor converts Ang II (angiotensin receptors, ATR1 & ATR2) to Ang 1–7 to prevent pro-inflammatory functions of Ang II. With ACE2 depletion, Ang II is overproduced, and in turn activates ADAM17. ADAM17 cleaves membrane-anchored proteins and immunological cytokines such as IL6, TNFα, and other ligands are released, followed by *trans*-signaling of IL6 through (soluble) IL6 receptor complex, which also mediates activation of JAK/STAT3. Similarly, AT1R-mediated inflammatory response is accompanied by other pathways as well *vis*-*a-vis* activation of NFkB-mediated cytokine storm generation, TNFα mediated activator protein 1 (AP1), mTOR-mediated NLRP3 expression, and apoptosis. Upregulation of *ZNF24* and *HIVEP1* (in Omicron) inhibits NFkB-mediated cytokine storms, thus preventing the development of ARDS. Moreover, expression of *MORC3*, *CNBP*, and *ZNFX1* regulates interferon type I response positively conferring a strong antiviral response. Furthermore, in response to previously explained activation of proinflammatory genes, *ZC3H12A* and *ZFP36* get recruited to bind with the mRNAs of TNFα and IL6, and direct them to their degradation (Figure 5D).

Consequently, it also indirectly stimulates the exhaustion of T cells to mediate the adaptive immune response. Henceforth, ZAPs and ZEB2 comes into play as positive regulators of T cell proliferation and survival and limit the functional degradation of exhausted T cells and help them release effector cytokines that would in turn stimulate cytotoxic T and B cells to release granules to kill the virus-infected cells. *ZSWIM1* is also required for T cell survival and therefore its downregulation can lead to inhibition of T cell-mediated adaptive immune response. Exhausted T cells are known to block further T cell activation affecting the adaptive immune response. However, the ZFPs come to rescue in Omicron and thus might provide a way for exhausted T cells to still be able to confer a viral clearance role (Figure 5D). Other ZFPs like, circHELZ and ZFYVEs also regulate immune response via pyroptosis and apoptosis. Activated mTOR pathway upregulates *BCL6*, *IL10*, and *HIF1A* expression, thereby increasing NLRP3 inflammasome activity, which is seen to be well regulated by circHELZ, generating an inflammatory response via pyroptosis. Moreover, upregulated ZFYVEs enhances apoptosis of virus-infected cells leading to reduced viral load (Figure 5D). All these DE ZFPs regulating signaling pathways at various levels suggest their nuanced but pivotal role in eliciting a broad and well-regulated immune response in Omicron. Contrarily, upregulated *MORC4* and downregulated *ZSCANs/ZKSCANs* in Pre-VOC is known to inhibit apoptosis, which might be contributing to virulence and enhanced pathogenicity.

## Discussion

The COVID-19 pandemic has marked the advent in NGS (Next Generation Sequencing) technologies, wherein it became indispensable to capture variations in SARS-CoV-2 genomes, leading to the identification of different lineages of the virus, with distinct epidemiological, immunological, and pathogenic properties. Emergence of variants of concern (VOC) of SARS-CoV-2 like Alpha and Delta revolutionized the entire outlook to this pandemic, since it brought along immense pressure on the health care system to curb disease progression as well as spread of infection due to devastating clinical course with enhanced mortality numbers. The possibility of a similar effect was expected during Omicron wave, but in spite of having huge numbers of mutations across SARS-CoV-2 genome as well as spikes, the clinical presentation by Omicron patients was largely mild. Several factors might have played a decisive role to this effect, vaccination being one of them, but our interest was in understanding this process from the host immune response perspective. Consequently, this study had been intricately framed to capture differences in the host response to infections with different variants of SARS-CoV-2: PreVOC, Delta, and Omicron, surging at different time points through the pandemic. Pre-VOC differentiated from wildtype or Wuhan strain with the presence of D614G mutation,^30^ wherein moderate presentation of clinical symptoms dominated, but the infection persisted way longer than observed during VOC times. Delta infections were severe leading to high rates of hospitalization and mortality,^28^^,^^31^ whereas Omicron infection displayed mild symptoms and enhanced clearance of viral load from the host.^32^^,^^33^ Our differential expression analysis between different variant groups revealed gene sets which corroborated with the clinical course of infection at different time points. The patients exhibited a robust and broad antiviral defense response to SARS-CoV-2 Omicron variant, with high expression of IFN genes, highlighting the induction of type I interferon response to Omicron.

Previous strains of SARS-CoV-2, be it Pre-VOC or VOCs like Delta, did not display such intense IFN response, which compromised the chances of the host to fight back the infection, leading to moderate/severe clinical manifestations. Earlier studies had reported that SARS-CoV-2 infection was characterized by a usual delayed interferon response relative to symptom onset, reflecting low innate antiviral defenses, leading possibly to peak virus replication and production of an enhanced inflammatory response that together facilitated inflammatory tissue damage and severity in COVID-19.^34^ Moreover, along with IFN activation, there was enhanced expression of both pro- and anti-inflammatory cytokines and chemokines during Omicron reflecting a balanced state of immune activation, which was previously missing during Pre-VOC and Delta infections. Notably, our data do suggest the presence of IFN III response genes (*IFNL1* and *IFNL3*) in Pre-VOC, which got all the more muted in Delta. Reportedly, it is well proven that Delta had progressively evolved over the ancestral variants to silence the innate immune response, thereby limiting cytokine and chemokine production as well as antigen presentation.^35^^,^^36^ Similar pattern of suboptimal expression of immune and stress response genes, observed in our Delta cohort well exemplified that Delta variant was capable of enhanced replication due to sustained suppression of host innate immune response, resulting in delayed or reduced intervention by adaptive immune system, which potentially contributed to severe symptoms and poor recovery.^35^

After looking at the overall host DE gene response to different variants of SARS-CoV-2, we tried to identify possible candidates which might be regulating the immune response to this effect. Studies have reported that Omicron viruses are less effective than Delta viruses in antagonizing the interferon response hence, may contribute to lower pathogenicity due to generation of robust IFN response in the host.^37^ Relevantly, an array of molecules such as proteins and non-coding RNAs, are known to contribute to SARS-CoV-2-host interactions, influencing the viral pathogenesis. Therefore, studying these interactions can be crucial for developing strategies and drugs to combat the infection. Interestingly, even a specific SARS-CoV-2 protein is delineated to study its interaction with host proteins. For instance, Zheng et. al. have studied the interactions of nucleocapsid protein with the host proteins.^38^ Consequently, viral host protein interactions provided substantial insights on differential regulation of host pathways by SARS-CoV-2 proteins between Pre-VOC, Omicron, and Delta. Multiple unique interactions were observed for both DEGs from VOC vs*.* Pre-VOC as well as Omicron vs. Delta, yet interestingly, majorly altered host immune functions which differentiated between the two comparison groups and diverged toward Omicron were those of IFN and innate immune response. One of the unique interactors for Omicron, N-LARP1, has been identified as a potent SARS-CoV-2 RNA binder displaying antiviral role by negatively influencing translation of SARS-CoV-2 RNAs to curb viral replication.^18^ Interestingly, it has been reported that SARS-CoV-2 proteins known to inhibit interferon response including S, nsp3, nsp6, nsp14, N, and M, are mutated in the Omicron.^37^ Docking studies with wildtype and mutated N proteins of Pre-VOC, Delta, and Omicron revealed the enhanced binding efficiency of LARP1 to SARS-CoV-2 N protein in Omicron, which could have potentiated the antiviral effects of LARP1. Moreover, the subjugated IFN response in Delta, might partially owe to its least binding energy and a stronger interaction with HNRNPA2B1. HNRNPA2B1, an RNA binding protein are activators of TBK1-IRF3 pathway for the induction of IFN-I and IFN-III expression, and pro-inflammatory cytokines production.^39^^,^^40^ Reportedly, agonists of HNRNPA2B1 dramatically ameliorated lung damage induced by SARS-CoV-2 Omicron (BA.1) infection in a hamster model and significantly suppressed viral infection.^22^ Importantly, the higher binding efficiency of N protein of Pre-VOC for HNRNPA3 and HNRNPA1 than wildtype provided an edge to the Pre-VOC variant to establish itself in the host population, as HNRP’s are known to positively regulate virus mRNA translation for effective establishment of viral infection.^41^

Through this study, we also highlighted the role of zinc finger proteins in modulating the antiviral IFN response and aiding in quick viral clearance during Omicron infection. The differential expression of a large number of ZFPs especially ZAPs in Omicron mechanistically support the induction of a strong type I IFN response, which together help the host to clear the infection in a short span of time. ZAPs belong to CCCH type zinc finger class which being RNA binders are known to restrict the replication of a number of RNA viral pathogens, including hepatitis B virus,^42^ alphaviruses,^43^ influenza A virus^44^ and retroviruses,^45^ and thus serve to control the severity of viral infections.^46^ During infection, it harbors propensity to bind CpG-rich viral RNA sequences and causes destruction of viral mRNAs,^47^^,^^48^^,^^49^ supporting viral clearance. Several studies have demonstrated that vaccination against infection with the Omicron variant elicited notably higher immune response compared to the other variants.^50^^,^^51^^,^^52^ This enhanced response might be attributable to the priming of specific T cells. Notably, our study has suggested a potential role of antiviral zinc fingers in supporting the exceptionally high T-cell-induced antiviral immune response, wherein T cell exhaustion, which has appeared as the primary mechanism underlying immune dysfunction in COVID-19,^53^ might plausibly be rescued by the ZAPs and ZEB2 in the Omicron infected patients. Thus, despite the evolution of the SARS-CoV-2 virus with enhanced immune evasion mutations and counteraction mechanisms, the host has eventually adapted to immune response activation patterns which could grossly interrupt the virus evolution delimiting the next phase of the COVID-19 pandemic.

SARS-CoV-2 infections has contributed toward evolved adaptability of the immune responses generated in the host, yet, it is important to understand whether COVID-19 positivity can alter the course of other viral infections such as dengue which warrants investigation due to yearly dengue endemic faced by many countries especially southeast Asia. Several studies have looked into the role of prior COVID-19 infection in dengue cases and understand cross-protective antibody response between the two viruses.^54^^,^^55^ One of the studies carried out in the children provided preliminary evidence that dengue fever might follow a less severe course in children with recent exposure to SARS-CoV-2 infection.^56^ Another study from India carried out an exploratory analysis on dengue occurrence and severity in the COVID-19 infected and vaccinated healthcare workers reporting that COVD-19 infection might cause symptomatic dengue rather than altering the severity (https://doi.org/10.1101/2023.01.09.23284366). Thus, partial immunity against COVID-19 may possibly exist in dengue-endemic regions.

### Limitations of the study

One limitation of our study is the constrained collection of clinical parameters. Due to the overwhelming workload faced by the clinicians and the challenges posed by the high infection rates during the COVID-19 pandemic, we were unable to collect a comprehensive set of clinical data. Notably, information regarding patient comorbidities, vaccination status, and the requirement for respiratory support was not present for all the patients in our cohort. These clinical parameters are critical for a comprehensive understanding of how the host immune response varies across different patient profiles and disease severities. It is important to note that our study samples were collected from a single hospital and a specific region, which provides the advantage of lower variability between the samples. To gain a more profound and inclusive understanding, future investigations should encompass multiple hospitals from diverse regions. Finally, we aim to carry out host response investigations beyond COVID-19 and explore other RNA virus infectious diseases, such as dengue (DENV-1, DENV-2, DENV-3, and DENV-4), which is highly pertinent as India is challenged with yearly dengue infections.

## STAR★Methods

### Key resources table

**Table:** 

REAGENT or RESOURCE	SOURCE	IDENTIFIER
**Critical commercial assays**		

Viral Transport Medium (VTM)	HiViral Transport Kit, HiMedia,	Cat. No: MS2760A-50NO
Viral RNA extraction	QIAmp viral mini kit, Qiagen	Cat. No. 52906
TRUPCR SARS-CoV-2 kit	3B BlackBio Biotech India Ltd	Cat. No. 3B304
PCR tiling of SARS-CoV-2 virus with Rapid barcoding	Oxford Nanopore	PCTR_9125_v110_revB_24Mar2021
LunaScript RT SuperMix	New England Biolabs	Cat. No. E3010L
SARS-CoV-2 specific primer pools	Integrated DNA Technologies	Product number: 10007184
rapid barcodes	Oxford Nanopore	SQK-RBK110.96
COVIDSeq	Illumina	Cat. No. 20043675
TruSeq® Stranded Total RNA Library Prep Gold	Illumina	Cat. No. 20020599
AMPure XP	Beckman Coulter	Cat. No. A63881
Agencourt RNAClean XP Kit	Beckman Coulter	Cat. No. A63987
Qubit dsDNA HS Assay kit	Symbio (Thermo Fisher Scientific)	Cat. No. Q32854
Agilent 2100 Bioanalyzer	Agilent	Cat. No. 5067-4626

**Deposited data**		

RNA-seq data	This study, NCBI Sequence Read Archive (SRA) database	BioProject ID: PRJNA676016 https://submit.ncbi.nlm.nih.gov/subs/sra/SUB8459696/overview, PRJNA678831 https://submit.ncbi.nlm.nih.gov/subs/sra/SUB8518381/overview (Pre-VOC), PRJNA868733 https://submit.ncbi.nlm.nih.gov/subs/sra/SUB11917851/overview, PRJNA952815 (VOCs) https://submit.ncbi.nlm.nih.gov/subs/sra/SUB13022295/overview

**Software and algorithms**		

Guppy	Basecalling (translating the electronic raw signal of the sequencer into bases), demultiplexing, adapter trimming.	version 2.1.0
Minimap2	Aligner used for alignment to the SARS-CoV-2 reference (MN908947.3). PMID: 29750242	V2.17
Nanopolish	Variant calling. PMID: 26076426	v 0.14.1
bcftools	To create consensus fasta. PMID: 33590861	v1.8
bcl2fastq	Basecall to standard compressed FASTQ file format	v2.19.0.316
FastQC	To determine the quality of raw reads.	v0.11.9
TrimGalore	Adapter and low-quality sequences filteration.	v0.32
HISAT2	Aligner used for alignment to the GENCODE human reference. PMID: 31375807	v 2.2.1
Samtools	Employed to convert, sort and index bam files. PMID: 33590861	v1.9
Salmon	To quantify transcript read abundance. PMID: 28263959	v1.10.1
Trimmomatic	Adapter and low-quality sequences filteration. PMID: 24695404	v0.40

**Software and algorithms**		

DESeq2	Differential gene expression analysis. PMID: 25516281	v1.38.3
EnhancedVolcano	Log fold change was plotted against p-adjusted value.	v1.16.0
clusterProfiler	Functional enrichment analysis	v4.8.3
ggplot2	Plotting and visualization	v3.4.2

**Other**		

Uniprot	Database for protein information and sequences.	https://www.uniprot.org/
KEGG	Database for pathway enrichment analysis	https://www.genome.jp/kegg/
Reactome	Database for pathway enrichment analysis	https://reactome.org/PathwayBrowser/
Enrichr	Web-tool for pathway enrichment analysis	https://maayanlab.cloud/Enrichr/
Cytoscape	Protein-protein interaction analysis	v3.9.1
PyMOL	In-silico mutagenesis on protein structure.	http://www.pymol.org/pymol
meta-PPISP server	Interaction sites prediction	https://pipe.rcc.fsu.edu/meta-ppisp.html
HADDOCK online server	Docking analysis. PMID: 26410586	v2.4; https://wenmr.science.uu.nl/haddock2.4/
Prodigy Webserver	Binding affinity prediction	https://wenmr.science.uu.nl/prodigy/
PDBsum	For interaction between two proteins.	https://www.ebi.ac.uk/thornton-srv/databases/pdbsum/
GraphPad Prism	Statistical analysis, plotting and visualization	v9.0
BioRender	For illustration and visualization	Lab License taken for BioRender
Inkscape	For illustration and visualization	v1.3

### Resource availability

#### Lead contact

Further information and requests for resources and reagents should be directed to and will be fulfilled by the lead contact, Rajesh Pandey (rajesh.p@igib.res.in).

#### Materials availability

This study did not generate new unique reagents and material.

#### Data and code availability

• RNA-seq data have been deposited at NCBI SRA, and are publicly available as of the date of publication. Accession numbers are listed in the key resources table. All the data reported in this paper will be shared by the lead contact upon request.
• This paper does not report original code.
• Any additional information required to reanalyze the data reported in this paper is available from the lead contact upon request.

### Experimental model and study participant details

#### Patient recruitment, sampling and data collection

The study cohort included a subset of 211 patients from a larger cohort of patients who were admitted to a tertiary care centre (Max Super Speciality Hospital, North India, Delhi, India) during different time-periods of the COVID-19 pandemic. Nasopharyngeal swabs were collected by the paramedical staff in Viral Transport Medium (VTM) (HiViral Transport Kit, HiMedia) on the day of hospital admission. The patients were confirmed COVID-19 positive by real-time reverse transcription-polymerase chain reaction assay (SARS-CoV-2 RT-PCR). The demographic and clinical details of the patients were collected from the electronic health record (EHR). All the patient samples were given anonymous barcodes at the CSIR-IGIB. SARS-CoV-2 whole genome sequencing was performed to assign the SARS-CoV-2 variants. The median age of the patients was recorded as *58 yrs* in the Pre-VOC, *64 yrs* in the Delta and *40*.*5 yrs* in the Omicron. The observed gender (M/F ratio) for the three subgroups Pre-VOC, Delta and Omicron is 80/44, 21/18 and 24/20 respectively. Taking infection with different SARS-CoV-2 variants and time points of infection into account, the patients were segregated into groups- a) Pre-VOC (n=125) which included samples from April 2020 to July 2020, and b) VOC (n=86) which included Delta and Omicron wave samples from April 2021 to July 2022. Institutional ethical clearance for the study was obtained from both CSIR-IGIB and Max hospital. The studies involving human participants were reviewed and approved by CSIR-IGIB’s Human Ethics Committee Clearance (Ref No: CSIR-IGIB/IHEC/2020-21/01). The patients/participants provided their written informed consent to participate in this study.

### Method details

#### SARS-CoV-2 whole-genome sequencing and data analysis

Genome sequencing was done using Oxford Nanopore Technology and Illumina Platforms. The ONT library preparation protocol- PCR tiling of SARS-CoV-2 virus with Rapid barcoding (Version: PCTR_9125_v110_revB_24Mar2021) was used for sequencing on ONT platform. In brief, 50 ng of total RNA was taken to synthesize single-stranded cDNA using LunaScript RT SuperMix (New England Biolabs), followed by viral genome amplification using SARS-CoV-2 specific primer pools (IDT). Further, the amplicons were pooled and ligated with rapid barcode sequences (SQK-RBK110.96) followed by SPRI bead purification. The purified library was ligated with adapter protein and loaded on the MinION Mk1B/Mk1C platform from Nanopore. Illumina sequencing library preparation was performed using COVIDSeq (Illumina) for the samples sequenced using Illumina sequencing platforms. The extracted RNA was utilized to synthesize cDNA and the viral genome was further amplified using two separate PCR reactions. The pooled amplicons then underwent tagmentation to tag adapters followed by the post-tagmentation clean-up and a second round of PCR amplification, ligating index adapters. The indexed amplicons were pooled, purified, and then quantified using Qubit dsDNA HS Assay kit (Thermo Fisher Scientific). A loading concentration of 11 pM was prepared by denaturing and diluting the libraries in accordance with the MiSeq System Denature and Dilute Libraries Guide (Illumina). Sequencing was performed on the MiSeq system, using the MiSeq Reagent Kit v3 (150 cycles) at 2×75 bps read length.

For data analysis, the raw fast5 files from ONT MinION were base called and demultiplexed using Guppy basecaller that uses the base calling algorithms of Oxford Nanopore Technologies (Nanopore Community) with phred quality cut-off score >7 on GPU-linux accelerated computing machine. Reads with a Phred quality score of less than 7 were discarded to filter the low-quality reads. The resultant demultiplexed fastq were normalised by a read length of 1200 bps (approximate size of amplicons) for further downstream analysis and aligned to the SARS-CoV-2 reference (MN908947.3) using the aligner Minimap2 v2.17.^57^ Nanopolish^58^ were used to index raw fast5 files for variant calling from the minimap output files. To create consensus fasta, bcftools v1.8 was used with normalised minimap2 output.

Fastqc was performed for all the raw fastq files generated from Illumina sequencing in order to check the Phred quality scores of all the sequences (Babraham Bioinformatics). A Phred quality score threshold of >20 was used for filtering reads from all the samples. Subsequently, adapter trimming was performed using the TrimGalore (Babraham Bioinformatics) and alignment of the sequences was performed using the HISAT2 algorithm^59^ on human data build hg38 to remove any human read contamination. Samtools v1.9 was employed to convert, sort, and index bam files. bcftools v1.8 was used to generate the consensus fasta using the unaligned/filtered reads, and variant calling was performed using the high-quality reads.

#### Human host RNA sequencing (RNA-seq) library preparation

RNA-seq libraries were prepared using Illumina TruSeq® Stranded Total RNA Library Prep Gold (Illumina) with 250ng total RNA isolated from the nasopharyngeal swabs of hospital admitted COVID-19 patients, as per manufacturer’s reference protocol. Cytoplasmic and mitochondrial rRNA were captured using biotinylated target-specific and removed with Ribo-Zero rRNA removal beads. Library preparation included double stranded cDNA synthesis, adenylation at 3′ends and ligation with index adapters. Subsequently, PCR based amplification was performed to enrich the cDNA libraries. PCR products were purified using AMPure XP beads (Beckman Coulter) and quantified using Qubit dsDNA HS Assay kit (Thermo Fisher Scientific). The quality of cDNA libraries was checked using the Agilent 2100 Bioanalyzer. Final loading concentration of 650 pM was used for sequencing, performed on the Illumina NextSeq 2000, with paired end 2×151 read length.

#### Data analysis: Quality control, mapping to reference and identification of differentially expressed genes

FastQC v0.11.9 was used to determine the quality of raw reads, followed by trimming of adapter sequences using Trimmomatic v0.40. Reads were mapped to human reference transcriptome (GENCODE) using Salmon quasi mapping tool to quantify transcript read abundance. Differential gene expression analysis was performed using DESeq2.^60^ To identify significant differentially expressed genes, Wald’s test with a cut-off of p-adjusted value of ≤ 0.05, and Log2 fold change of ≥ ± 2 was applied. Differential gene analysis was carried out between the groups, a) VOC and Pre-VOC, b) Delta and Pre-VOC, c) Omicron and Pre-VOC, and d) Omicron and Delta. Log fold change was plotted against p-adjusted value using EnhancedVolcano R package (https://github.com/kevinblighe/EnhancedVolcano).

#### Pathway enrichment analysis

Functional enrichment of DEGs were performed using clusterProfiler package^61^ based on pathways and terms from the Kyoto encyclopedia of genes and genomes (KEGG) database. Statistical significance of the pathways was calculated using Fisher’s Exact test. Pathways related with any infection and immune signalling, and with a *p value* cutoff of 0.05 were considered. The pathways were plotted using the ggplot2 R package, against the combined score and number of genes involved in the pathways.

#### SARS-CoV-2 human host protein-protein interaction

For significant combined Differentially Expressed Genes (DEGs) list, protein-protein interaction (PPi) analysis was carried out using interactome of SARS-CoV-2 and human proteins published by Gordon et al., Li et al., Stukalov et al. and Zhou et al.^62^^,^^63^^,^^64^^,^^65^ in the Cytoscape v3.9.1. The pathway enrichment analysis has been applied with each interactor specific to the SARS-CoV-2 proteins to highlight the statistically enriched pathways associated with the corresponding interactions seen across the groups of Pre-VOC, Delta and Omicron using Enrichr searched through Reactome database. For each pathway term, a hypergeometric test with Benjamini-Hochberg correction was performed with a corrected p-value < 0.05.

#### Protein-protein complex docking

To comprehend the differential effects of distinct variant interactions with the host transcriptome signatures, host-pathogen protein-protein docking was performed. The significant differentially expressed genes that were SARS-CoV-2 N protein interactors, was taken downstream for the binding analysis. The full-length structure and the full-length amino acid sequence of SARS-CoV-2 N protein was taken from Uniprot (id: P0DTC9) and modelled using Alpha Fold. Individual mutation analysis and subsequent Fisher test was carried out to obtain N protein mutations significant to Pre-VOC, Delta and Omicron. Further, these mutations were imparted in the N-protein using mutagenesis of pyMOL software (Schrödinger L, DeLano W. PyMOL [Internet], 2020, available from (http://www.pymol.org/pymol). The structures of host proteins interacting with the SARS-CoV-2 N protein were directly taken as Alpha Fold structure from Uniprot. Furthermore, binding sites for all the proteins were taken from meta-PPISP server (https://pipe.rcc.fsu.edu/meta-ppisp.html) where active residues are predicted using neural-network algorithm (Table S4). Subsequently, the docking analysis was performed using HADDOCK online server.^66^ In addition, binding energy and disassociation constants of docked complexes were taken from the haddock’s prodigy, and the interactions were examined using PDBsum (https://www.ebi.ac.uk/thornton-srv/databases/pdbsum/). RMSD value of predicted and experimental structure of N protein (PDB id:6M3M) was observed to be 0.5, whereas, all the obtained RMSD values were observed to be less than 2 for the host protein structures.

### Quantification and statistical analysis

Comparison between COVID-19 patient groups were described using descriptive statistics, which display continuous variables as medians or interquartile ranges and categorical variables as percentages or proportions. Wherever appropriate, we compared the differences using the Mann–Whitney *U* test (Figures 1B-iv-vii), Kruskal Wallis test and Chi-square testing (‘∗’, ‘∗∗’, ‘∗∗∗’ signifying p-value <0.05, <0.01, <0.001 respectively) (Table 1). To identify significant differentially expressed gene (DEGs), Wald’s test was applied (Figure 2A). Statistical significance calculation of pathways obtained with KEGG database was done using Fisher’s Exact test with a *p value* cutoff of 0.05 (Figure 2C). For Enricher based pathway analysis for protein-protein interaction studies, a hypergeometric test with Benjamini-Hochberg correction was performed with a corrected p-value <0.05 (Figure 3). Statistical significance of the mutations were calculated using Fisher’s exact test (Figure 4). The statistical analysis was performed using GraphPad Prism v9.0 and R v4.0.2 available from CRAN or Bioconductor. Most of the graphs and illustrations were produced using the R package ggplot2 (https://cran.r-project.org/web/packages/ggplot2/index.html) and BioRender.

## Supplemental information

Document S1. Figures S1 and S2 and Tables S1, S2, and S4

Table S3. Mutations analysis across the three groups – Pre-VOC, Delta, and Omicron and statistical significance calculation through Fisher’s test, related to Figure 4
