# Supplementary material for: Co-evolution of SARS-CoV-2 variants and host immune response trajectories underlie COVID-19 pandemic to epidemic transition
Source: iScience. 2023 Oct 27;26(12):108336. doi: 10.1016/j.isci.2023.108336 (PMC10663816; doi:10.1016/j.isci.2023.108336)
Supplement: Document S1. Figures S1 and S2 and Tables S1, S2, and S4 [file mmc1.pdf]

iScience, Volume *26*

Supplemental information

Co-evolution of SARS-CoV-2 variants and host immune response trajectories underlie COVID-19 pandemic to epidemic transition

Ranjeet Maurya, Aparna Swaminathan, Uzma Shamim, Smriti Arora, Pallavi Mishra, Aakarshan Raina, Varsha Ravi, Bansidhar Tarai, Sandeep Budhiraja, and Rajesh Pandey

Table S1: The overlapping interactions of differentially expressed host genes and SARS-CoV-2 proteins across the groups Pre-VOC, Delta and Omicron. Related to Figure 3.

VOC vs Pre-VOC	SARS-CoV-2 genes
BRD2	E
ATP1A1	M
CTSB	M
NFE2L2	M
TNFAIP2	M
WDFY3	M
HSPA1A	N
PRKAR1A	nsp10
MYCBP2	nsp12
GAPDH	nsp13
CLTC	nsp13
LDHA	nsp13
NIN	nsp13
PGK1	nsp13
NCF2	nsp15
VIM	nsp15
AHNAK	nsp16
PFKFB3	nsp16
EIF4G2	nsp2
CSDE1	nsp2
EEF1A1	nsp2
EIF4A1	nsp2
FKBP15	nsp2
HDLBP	nsp2
IQGAP1	nsp2
FCER1G	nsp3
SSH2	nsp3
HLA-C	nsp4
ATP13A3	nsp6
MSN	nsp6
VMP1	nsp6
GNB1	nsp7
RHOA	nsp7
SDCBP	nsp7
H3-3A	nsp8
TPI1	nsp8

VASP	nsp8
ALDOA	nsp9
PKM	nsp9
CTSD	ORF10
ANXA5	ORF3
B2M	ORF3
CD44	ORF3
ECE1	ORF3
GNA13	ORF3
HLA-E	ORF3
IFITM1	ORF3
IFNGR1	ORF3
NCOA4	ORF3
NOTCH2	ORF3
PTPRJ	ORF3
SLC6A6	ORF3
SPAG9	ORF3
CPD	ORF3a
HLA-A	ORF3a
KIF5B	ORF6
NUP98	ORF6
CCNL1	ORF7a
DOCK5	ORF7a
RNF213	ORF7a
CANX	ORF7b
SLC38A2	ORF7b
CALR	ORF8
HSP90B1	ORF8
PLAU	ORF8
SQSTM1	ORF8
UBR4	ORF8
MYH9	ORF9c

Table S 2: The unique interactions of differentially expressed host genes and SARS-CoV-2 proteins across the groups Pre-VOC, Delta and Omicron. Related to Figure 3.

VOC vs Pre-VOC	SARS-CoV-2 genes	Omicron vs Delta	SARS-CoV-2 genes
RPL39	E	RPL13	E
ACADSB	M	SLC45A1	M
ATP7B	M	ATP6V1A	M
L2HGDH	M	IRAK1	M
MCFD2	M	PRKDC	M
MT-CO2	M	RTN4	M
ORC4	M	SPG11	M
ELAVL1	N	STOM	M
HNRNPA1	N	H1-2	N
HNRNPA2B1	N	LARP1	N
HNRNPA3	N	RPS4X	N
HSPA4	N	RPS6	N
RPS24	N	RPS8	N
DDX60	nsp1	RPS9	N
GORASP1	nsp13	EFHD2	nsp10
CEP350	nsp13	ERGIC1	nsp10
GOLGB1	nsp13	SVIL	nsp10
MIPOL1	nsp13	ZYX	nsp10
PDE4DIP	nsp13	ACTN1	nsp12
REL	nsp14	CORO1C	nsp12
EIF2S2	nsp15	FERMT3	nsp12
BCLAF1	nsp2	PPP1R18	nsp12
ITPRID2	nsp2	TLE3	nsp13
SET	nsp2	CNBP	nsp14
HMGN2	nsp3	LMNB1	nsp15
ALG11	nsp4	SH3BGRL3	nsp15
AP3B1	nsp5	WASHC4	nsp2
PIGU	nsp6	P4HB	nsp4
TMEM41A	nsp6	PDIA3	nsp4
MT-ATP6	nsp6	PPIB	nsp4
MT-ND4	nsp6	RPLP2	nsp5
RAB7A	nsp7	ADPGK	nsp6
NDUFAF2	nsp7	ATP6AP1	nsp6
MRPS27	nsp8	COTL1	nsp6
ETF1	nsp9	DHCR24	nsp6
ITM2B	ORF3	TMEM154	nsp6
KYNU	ORF3	RAB1A	nsp7

MYOF	ORF3	HECTD1	nsp8
PCDHA4	ORF3	STAT6	nsp9
SORD	ORF3a	S100A9	ORF10
PCM1	ORF6	ADAM17	ORF3
GPAT4	ORF7b	NAPA	ORF3
TMED10	ORF7b	RAB31	ORF3
VTI1A	ORF7b	RNF149	ORF3
ZDHHC6	ORF7b	SLC12A6	ORF3
APOOL	ORF7b	TNFRSF1A	ORF3
ASPH	ORF8	TNIP1	ORF3
HSPA5	ORF8	CAP1	ORF3a
ZNF274	S	MGAT1	ORF3a
TPM3	S	ATP5F1B	ORF3b
		CYRIB	ORF3b
		ITPR2	ORF6
		TANK	ORF6
		TPR	ORF6
		TOM1	ORF7a
		USP34	ORF7a
		CKAP4	ORF7b
		CTNNA1	ORF7b
		IGF2R	ORF7b
		PLIN2	ORF7b
		SLC15A4	ORF7b
		TCIRG1	ORF7b
		VSIR	ORF7b
		ZDHHC18	ORF7b
		OS9	ORF8
		MACROH2A1	ORF9b
		MYL12A	ORF9c
		MYL6	ORF9c
		RPS19	ORF9c

Table S4: Binding sites of the host genes for SARS-CoV-2 N protein from meta-PPISP server. Related to Figure 4.

**Protein**	**Active residue**
ELAV1	201-232
HNRNPA3	221-224,229-237,241-243,257-312,342-352,361-366,371-373
HNRNPA1	202-204,206-223,226-256,258,260,263,265,267,273,274,279-281,284-288,339-359
HNRNPA2B1	218,219,234,237,239-250,261,263,266-300,311,313-338,349
RPS4X	1,2,3,4,5,6,7,8,10,12,13,14,25,26,27,29,30,32,38,81
RPS6	61,96,135,143,145,156,209,210,212,213,216,217,224,231
RPS8	1,2,3,4,5,6,7,8,9,18,20,21,22,23,24,25,26,27,28,29,30,31,47,49
RPS9	1,2,3,4,5,6,7,8,9,10,11,12,13,14,41,45,46,48,49,50,52,53,56,69,72,73,75,76,77,78,79
HSPA4	1,9,12,14,19,20,22-24,27,31-33,34,168,184,367,369,374,382,383,385-389,833,836-839
RPS24	9-22,24,48,72,77,81
H1-2	1-8,10,13,14,16,19-22,210,212,213
LARP1	1-6, 353-388, 833,860-865
N Protein	211-220,222,223,226,227,230,307,309-323,330-337,339

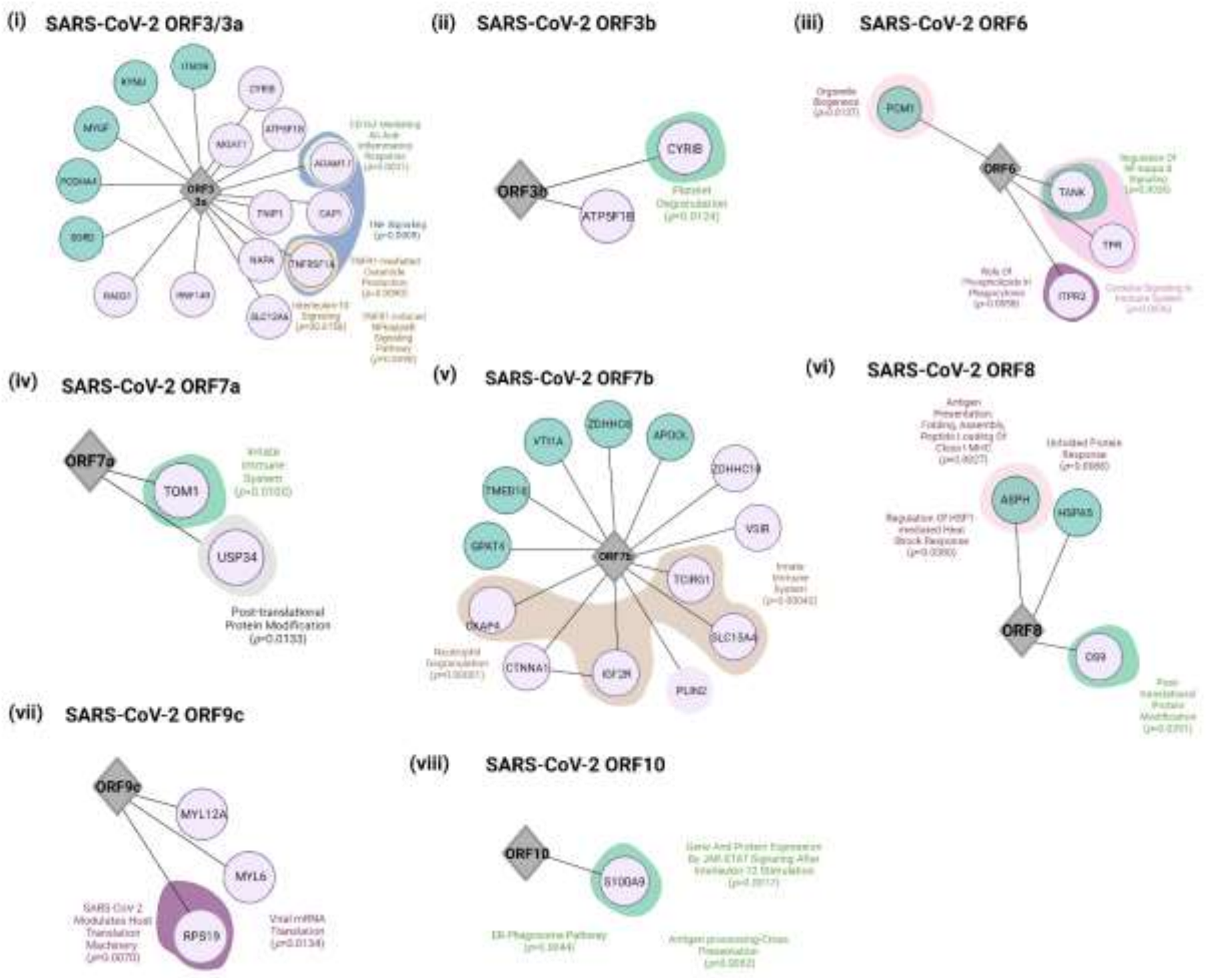

Figure S1: SARS-CoV-2 host protein-protein interactome. Among the SARS-CoV-2 proteins, orf3a (i) orf6 (iii) orf7a (iv) orf7b (v) displayed interactions with genes that were involved in innate immune system pathways such as NF-KB activated and signal survival, TNF signalling, interleukin 10- signaling, innate immune system, neutrophil degranulation particularly abundant in the group Omicron *vs* Delta. Orf3b (ii) showed significant enrichment for platelet degranulation, whereas orf8 (vi), orf9c (vii) and orf10 (viii) displayed post translational and SARS-CoV-2 modulation of host in Omicron. Related to Figure 3.

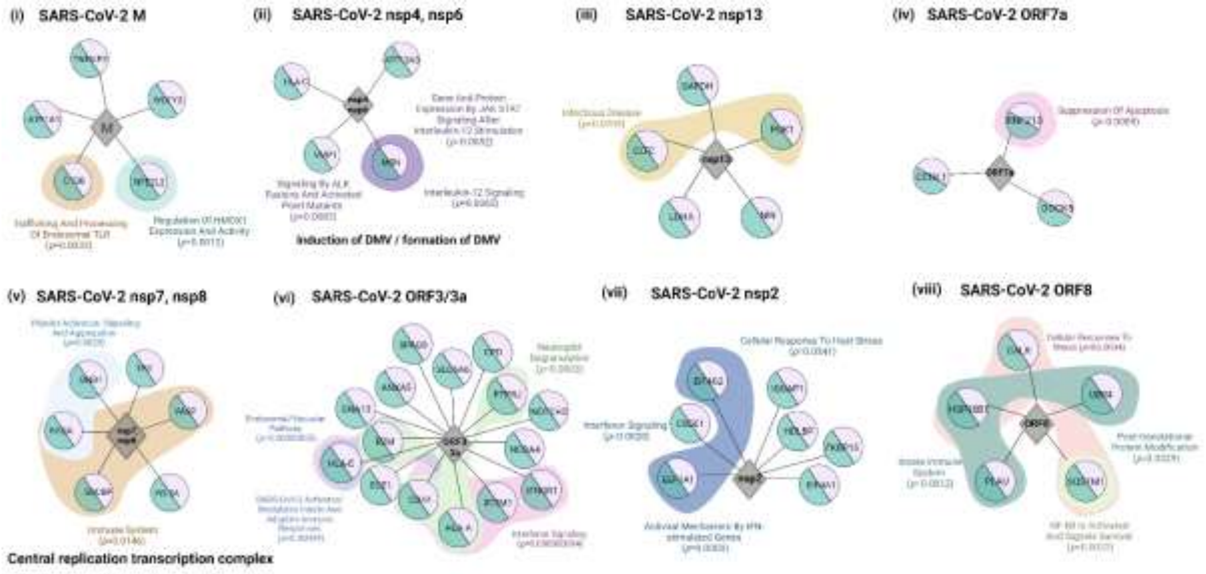

Figure S2: SARS-CoV-2 host protein-protein interactome. Overlapping SARS-CoV-2 – host protein-protein interactions (i) M nsp2 (vii), M, nsp13 (iii) and orf8 (viii) orf3a (vi) were found to have large number of interactions. Together, the key pathways that were enriched for the common host proteins interacting with SARS-CoV-2 proteins were protein trafficking, endosomal pathways, interleukin signaling, NF-KB signaling, neutrophil degranulation, cellular response to stress pathways, highlighting the prompted immune response to SARS-CoV-2 by the host post infection. Related to Figure 3.
